# Computational Evolutionary Methodology for Knowledge Discovery and Forecasting in Epidemiology and Medicine

**DOI:** 10.1063/1.2937609

**Published:** 2008-05-08

**Authors:** Dhananjai M. Rao, Alexander Chernyakhovsky, Victoria Rao

**Affiliations:** a CSA Department, Miami University, Oxford, OH 45056, USA; b William Mason High School, Mason, OH 45040, USA; c Cybernetic Evolution Inc., Mason, OH 45040, USA; IAENG Secretariat and Oxford University Computing Laboratory; California State University; University of Florida

## Abstract

Humanity is facing an increasing number of highly virulent and communicable diseases such as avian influenza. Researchers believe that avian influenza has potential to evolve into one of the deadliest pandemics. Combating these diseases requires in‐depth knowledge of their epidemiology. An effective methodology for discovering epidemiological knowledge is to utilize a descriptive, evolutionary, ecological model and use bio‐simulations to study and analyze it. These types of bio‐simulations fall under the category of computational evolutionary methods because the individual entities participating in the simulation are permitted to evolve in a natural manner by reacting to changes in the simulated ecosystem. This work describes the application of the aforementioned methodology to discover epidemiological knowledge about avian influenza using a novel eco‐modeling and bio‐simulation environment called SEARUMS. The mathematical principles underlying SEARUMS, its design, and the procedure for using SEARUMS are discussed. The bio‐simulations and multi‐faceted case studies conducted using SEARUMS elucidate its ability to pinpoint timelines, epicenters, and socio‐economic impacts of avian influenza. This knowledge is invaluable for proactive deployment of countermeasures in order to minimize negative socioeconomic impacts, combat the disease, and avert a pandemic.

### MOTIVATION

Communicable and vector-borne diseases, primarily induced by viral and bacterial infections, are the most common forms of ailments amongst humans and domesticated farm animals [1]. Despite numerous advances in epidemiology and medicine many of these diseases continue to defy human efforts to treat and control them [1]. The source of such resistance to treatment and control is two fold. First, the incessant evolutionary processes such as genetic reassortment, recombination, and mutation induce changes to organisms, thereby constantly morphing their antigenic characteristics. Second, the vectors of the disease, such as birds and animals, often disperse the pathogens over wide areas. The scenario is further convoluted due to complicated symbiotic processes between the vectors and other organisms including humans. Rapid changes in phylogeny and bio-diversity pose significant challenges in vaccine manufacturing. In addition, socio-political restrictions and technological limitations further magnify the challenges involved in controlling intercontinental spread of infectious diseases.

CP1007, *Current Themes in Engineering Technologies*, edited by S.-I. Ao, M. A. Amouzegar, and S.-S. Chen
© 2008 American Institute of Physics 978-0-7354-0526-4/08/$23.00

The aforementioned issues are salient characteristics of avian influenza which is caused by H5N1, a highly virulent strain of the influenza-A virus [2, 3, 4]. In Spring 2006, it was established that infected migrating waterfowl causes transcontinental dispersion of H5N1. The virus has a devastating impact on poultry causing 100% mortality within 48 hours of infection [5]. Currently, mass culling of poultry is the recommended approach to contain the disease and this aspect causes significant economic hardships [6]. Moreover, the pathogen also spreads to humans through direct contact with infected poultry and contaminated surfaces [5]. Many researchers have stated that H5N1 has the potential to cause the next pandemic [5, 7].

Proactive administration of suitable vaccination is the prevention mechanism for influenza [2, 5]. Unfortunately, a myriad of technological and socio-political issues have rendered manufacturing and distribution of H5N1 vaccine a significant challenge [2, 5]. Some of these issues include: *(i)* rapid mutations in the virus make it practically infeasible to predict antigenic characteristics for manufacturing vaccines; *(ii)* current vaccine manufacturers do not have sufficient capacity; and *(iii)* targeted distribution of limited quantities of the vaccine is a challenge [5, 8]. These issues necessitate that preventing and controlling avian influenza requires proactive international coordination and strategic deployment of countermeasures. The effectiveness of proactive strategies, including those proposed by the World Health Organization (WHO), will greatly benefit from prediction and forecasting of epicenters and timelines of H5N1 outbreaks. However, forecasting and proactive deployment of countermeasures requires global knowledge about the current and future epidemiology of the disease.

Analogous scenarios apply to current and future communicable and vector-borne diseases as well. Humanity needs to be prepared to proactively and effectively combat emergent diseases to minimize loss of human lives and impacts to global economy. Combating disease requires extensive knowledge about epidemiology, vaccinal characteristics, epicenters of disease outbreaks, timelines, economic impacts, and human mortality. Accordingly, we propose a computational evolutionary methodology to address the aforementioned need.

### PROPOSED APPROACH

One of the most promising methodologies to rapidly analyze a complex, stochastic system consisting of millions of organisms is bio-simulation [9, 10]. Bio-simulations have shown to be intuitive, realistic, and provide close reflection of epidemiological characteristics [10, 11]. The proposed approach aims to leverage bio-simulations to perform epidemiological analysis through the use of evolutionary eco-models of selected organisms in a global ecosystem. In the eco-models individual organisms are modeled (typically as smart agents) to evolve in a virtual ecosystem [12]. As the ecosystem evolves, necessary attributes at different levels of resolution are recorded to infer various characteristics. Note that in this evolutionary approach the behaviors of organisms (modeled as smart agents) are not coerced. Instead they are permitted to proceed "naturally" reacting to changes in the ecosystem and reflecting their real world counterparts. Once verified, the evolutionary bio-simulations can be used to further analyze a wide variety of scenarios to discover more comprehensive knowledge.

### Background on Computational Evolutionary Approaches

Computational evolutionary methodology employs computers and digital models to study and analyze evolution, phylogeny, and associated biological characteristics of organisms [12, 13, 14]. This methodology includes a wide variety of other modeling, simulation, and analysis techniques as well [12, 13, 14]. Since our proposed methodology uses bio-simulations to analyze the natural progression and evolution of avian influenza, it falls under the category of computational evolutionary methods.

The fundamental motivation for computational approaches, that enable study organisms in silico rather than in vitro, stems from many of their advantages [12]. The primary advantage is that existing knowledge can be used to create virtual organisms, rapidly and economically analyze numerous future generations, and draw inferences [12, 13, 14]. Computational evolutionary approaches have shown to be excellent candidates for analyzing phylogeny of microorganisms such as bacteria and viruses [12]. These approaches are gaining significant importance in biology and medicine.

### Epidemiology of Avian Influenza

The proposed computational evolutionary methodology can be applied to a broad range of biological systems [12, 13, 14]. However, here we will focus on the issues involved in applying the proposed methodology to study and analyze the epidemiology of avian influenza along with its socio-economic impacts. The objective is to use existing information on various organisms in the ecosystem, namely migrating waterfowl, poultry, and humans, to discover additional knowledge for forecasting timelines and epicenters of disease outbreaks. The motivation is to empower disease control centers and emergency management agencies with crucial information required to contain and control outbreaks to save human lives and minimize socio-economic impacts.

## SEARUMS: A COMPUTATIONAL EVOLUTIONARY ENVIRONMENT FOR AVIAN INFLUENZA

Realizing the advantages of the proposed computational, evolutionary methodology requires the use of an effective software environment for eco-modeling, bio-simulation, and epidemiological analysis [10]. Specifically, in this case, the software environment must be conducive for analyzing the epidemiology of avian influenza. The software environment must facilitate rapid modeling and simulation (M&S) to minimize analysis time frames, thereby accelerating deployment of countermeasures. Moreover, the software must be portable and accessible to enable its widespread use [10]. Accordingly, we have endeavored to design and develop such a software environment called SEARUMS.

SEARUMS is an acronym for Studying Epidemiology of Avian Influenza Rapidly Using Modeling and Simulation. It is an Java-based, integrated, graphical modeling, simulation, and analysis environment that is specialized for epidemiological study of avian influenza. SEARUMS provides an extensible, agent-based, spatially explicit modeling front-end coupled with a discrete event simulation kernel. The conceptual model of the software agents has been developed from corresponding Markov processes that provide a strong mathematical grounding. SEARUMS has been integrated with libraries for plotting graphs and charts that ease visualization of simulation results and aid in analysis.

In addition, SEARUMS includes comprehensive models that incorporate real-world statistical data on: waterfowl migration, published by Global Register Of Migratory Species (GROMS) [15]; waterfowl species that are at higher risk to carry the virus [6]; global poultry population and distribution, published by the Food and Agriculture Organization (FAO) of the United Nations [16]; and human population in metropolitan areas of the United States, obtained from the U.S. Census Bureau [17]. The model is stored as a portable XML document that can be readily reused and further extended. This aspect alleviates many procedural hurdles and promotes sharing and collaboration. SEARUMS is envisioned to serve as a global, multi-disciplinary environment that seamlessly integrates knowledge from various fields so that epidemiologists, economists, and disease control centers can collaboratively use it to combat avian influenza.

### Conceptual Epidemiological Model

The epidemiology of avian influenza currently involves three primary types of entities, namely: migrating waterfowl flocks, humans, and poultry [6, 8]. Migrating waterfowl are the primary vectors of the H5N1 virus [18]. They indirectly transmit the virus to poultry in their vicinity through infected food, water, and feces [19, 20]. The disease is transmitted to humans that come in direct contact with contaminated surfaces or infected poultry [21]. In other words, the transmission of infections is determined both by space and time. Consequently, an epidemiological model needs to incorporate temporo-spatial interactions between the aforementioned three entities. Accordingly, we have developed our conceptual model using spatially explicit, discrete-time Markov processes as described further below [22]. Note that, since the goal of this section is to present factors steering the design and implementation of SEARUMS, it does not delve into mathematical details that are available in the literature [10].

The proposed conceptual model consists of a set of interacting entities. An entity may represent a flock of waterfowl, poultry, or confederation of humans. Each entity is mathematically modeled by a corresponding Markov process. A Markov process undergoes instantaneous, probabilistic state transitions to reflect the behavior of its real-world counterpart [10]. Temporal state transitions induce changes to state variables, which in this case include population counts, location, infection percentage, and migration attributes. These changes corresponding various conditions such as: migration, healing or regeneration, and death or reduction in population. In conjunction with temporal changes, spatial interactions between entities are also captured as state transition probabilities. The spatial interactions, that occur in a spatially explicit models, have been mathematically modeled using principles of Euclidean geometry [23]. The surface of the earth is represented as an Euclidean plane and each entity has a circular area of influence. Interactions between entities occur when their circle of influence overlaps. Interactions between entities and the degree of overlap are used to model the intensity and spread

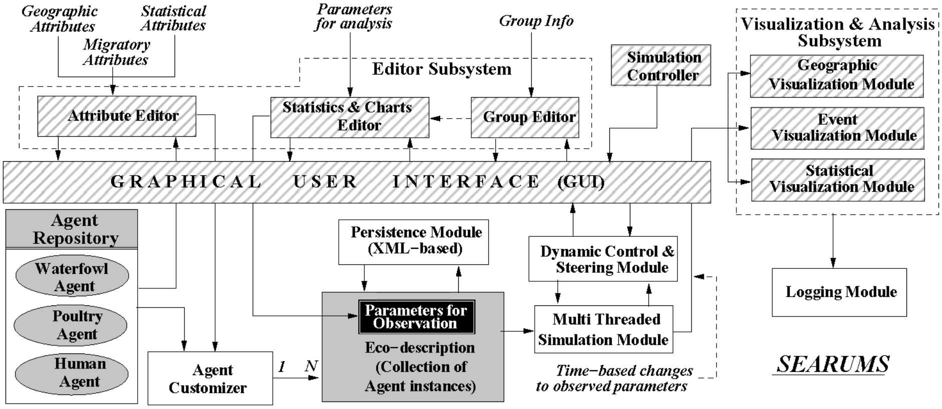

**FIGURE 1.** Architectural overview of SEARUMS. GUI modules are highlighted with a striped background. SEARUMS can be downloaded from `http://www.searums.org`.

of infection. Such a modeling approach is widely used in spatially explicit ecological models to reduce computational complexity [23, 24, 25].

In SEARUMS, each Markov process in the aforementioned mathematical formalism is modeled as a smart agent [24]. The simulation consists of a collection of interacting smart agents whose temporal behavior is causally coordinated using a discrete event kernel. The state has been implemented using a set of attributes that incorporate both temporal and spatial variables. Note that our methodology does not coerce behaviors or interactions between entities. Instead, each entity mimics the behavior of their real-word counterpart which significantly simplifies implementation, reduces computational complexity, enables lucid use of real-world data, and eases addition of new instances including new types of software agents.

### ARCHITECTURE OF SEARUMS

SEARUMS is an eco-modeling and bio-simulation virtual environment that uses a computational evolutionary methodology for study and analysis of epidemiology of avian influenza. It has been developed in Java by capitalizing on many of its object oriented programming features [26]. SEARUMS is designed to be a user friendly, integrated, graphical modeling, simulation, visualization, and analysis environment for conducting epidemiological analysis of avian influenza using an Agent-based, Spatially Explicit (ASE) model. These design goals have been achieved by composing the system using a collection of interdependent but loosely coupled modules. Each module has a well defined functionality that can be accessed and utilized via a set of Application Program Interface (API) method calls. API of the modules are Java interface classes that are implemented by each module. Interactions between modules is achieved through interface classes to ensure loose coupling. This approach permits seamless "plug and play" of modules and the environment is composed by loading suitable modules dynamically on-demand via Java's reflection API [26]. Such an implementation approach has been adopted to ease customization and extension of SEARUMS without requiring changes to its design or impacting existing modules.

An architectural overview of the modules constituting SEARUMS is shown in Figure 1. The modules can be broadly classified as core modules and Graphical User Interface (GUI) modules. The core modules of SEARUMS are the Agent Repository, Agent Customizer, Persistence Module, Dynamic Control & Steering Module, Simulation Module, and Logging Module. These modules provide the core M&S functionality of SEARUMS. The GUI facilitates interactions with the core modules via convenient and intuitive user interfaces. The GUI modules can be further categorized into the Editor subsystem, the Simulation Controller, and the the Visualization & Analysis subsystem. SEARUMS uses the Model-View-Controller pattern to couple the core modules, the GUI models, and the Eco-description. The design permits the GUI modules to be easily replaced with a minimal command-line text interface for running SEARUMS in offline batch mode. The batch mode is useful for performing repeated runs or analyzing different scenarios on computational clusters.

The modules and subsystems constituting SEARUMS cooperatively operate on a shared, in-memory representation of the model called the Eco-description. The Eco-description is a centralized data structure that includes all the information necessary for modeling, simulation, and analysis. It is composed using a collection of Java classes and that provide efficient access to data and information required by the various modules. The primary information encapsulated by the Eco-description relates to the smart agents [24] that constitute the model. As shown in Figure 1, the agents are organized into an `Agent Repository` to facilitate instantiation and use via Java reflection API. Currently, SEARUMS includes the following three smart agents: `Waterfowl Agent` that represents a migrating waterfowl flock, `Poultry Agent` that models behavior of poultry flocks, and `Human Agent` that models humans. Each agent has its own behavior that reflects the characteristics of its real-world counterpart. The behaviors are customized to represent specific instances of an agent by specifying suitable values for the exposed attributes via the `Attribute Editor` GUI module. The attributes of an agent include:

1. *Geographic attributes* that indicate the location (latitude and longitude) and logical association with countries and continents. In addition, each agent has a circle or influence that circumscribes its neighborhood.
2. *Migratory attributes* are specified only for agents whose location changes over the lifetime of the simulation. The migratory attributes are described as a sequence of migration points. Each migration point has geographical and chronological (arrival and departure dates) attributes associated with it. In SEARUMS, only one complete migration cycle needs to be specified. The software automatically reuses the information to simulate annual migratory cycles.
3. *Statistical attributes* for agent instances include their initial population, density and distribution, initial infection percentage, infection spread parameters, incubation periods, mortality rates, and population regrowth parameters.

The agents implement the conceptual, mathematical model of the system developed using Markov processes. They are added to a model via suitable toolbar buttons or menu options provided by SEARUMS. Agent instances are created with default attributes from the Agent Repository by the `Agent Customizer` module using Java's reflection API. Once instantiated, the attributes for agents can be modified via the `attribute editor` module. The agents are implemented as a family of Java classes by extending a common base class called `Agent`. The `Agent` class provides methods for interacting with the simulation kernel, inspecting the neighborhood, scheduling events, and interfacing with the GUI modules.

The agents in a model are logically organized into hierarchical sets called groups. SEARUMS permits multiple top-level groups with an arbitrary number of hierarchies, with one or more sub-groups at each hierarchical level. An agent can be a member of multiple groups. The groups serve several different purposes in SEARUMS. A group can be used as a parameter for statistical analysis and for plotting charts. For example, a group called "United States" can be created with 50 different sub-groups, one for each state, encompassing various agents. The main "United States" group can be selected for plotting charts and SEARUMS automatically collates and plots data for each state. Note that even though graph plotting is restricted to one hierarchical level, statistics for plotting are collated in a recursive, depth-first manner and includes data from all agents in underlying hierarchies. A modeler can use a combination of groups to perform multi-faceted analysis at different scales. In addition, groups can be included or excluded from simulations for analyzing different scenarios. The GUI modules utilize groups to provide control on visibility of agents to manage details displayed on the screen. The Group Editor module provides the user interface for managing group entries and hierarchies.

Once all the agent instances and groups have been established in a model, the parameters for observation are added to the Eco-description. These parameters are selected by the user via the Statistics & Charts Editor from a list of options. The list includes the attributes of the agents and the groups in the Eco-description. Each parameter is configured to be sampled hourly, daily, or weekly in terms of simulation time. Moreover, each parameter can be subjected to statistical operations, such as sum, mean, and median. SEARUMS can dynamically (*i.e.,* during simulation) plot and save a variety of charts including: line graphs and pie charts. Multiple charts can be simultaneously used for analyzing a variety of data.

All of the aforementioned information is stored as an integral part of the Eco-description. The Eco-description can be saved for future reuse via the `Persistence Module`. The Eco-description is unmarshalled into an XML document that is compliant with a predefined XML schema. Serializing to an XML document has its advantages. First, it enables simple scripts to be developed that can modify specific values and perform multiple simulation runs in batch mode. Second, XML documents can be readily version controlled and archived using commonly available revision control systems like CVS and Subversion. Third, it eases documentation, validation, sharing, and reuse of valuable domain-specific statistical data collated by different researchers from diverse sources. Such features play an important role in facilitating large-scale, collaborative epidemiological studies.

The `simulation module` performs the task of conducting a Discrete Event Simulation (DES) using the Eco-description. This module utilizes a multi-threaded DES kernel that manages and schedules the discrete events generated by the Agents. Multi-threading enables the DES kernel to exploit the compute power of multi-processor or multi-core machines thereby reducing the wall-clock time for simulation. The number of threads spawned by the DES kernel is configurable. Each thread processes concurrent events (events with the same timestamp) in parallel without violating the causal constraints between events. The Dynamic Control and Steering Module provides the infrastructure to control the DES kernel. In addition, it permits selected agent attributes to be modified during the course of simulation. The Simulation Controller module provides the graphical interface to the Dynamic Control & Steering Module.

### ECO-MODEL FOR VERIFICATION AND USE OF SEARUMS

The initial experiments conducted using SEARUMS centered around its verification and validation. For this purpose, we have developed an Eco-description using selected, high risk species of waterfowl as reported by Hagemeijer *et al* [27]. Table 1 lists the waterfowl species, including high risk species [27], used in the model. The migratory flyways of the waterfowl and their population has been collated from data published by various organizations [2, 4, 15, 16, 17, 27]. For modeling and simulation purposes the dates for migration were approximated to the middle of the months reported in the statistics. The initial positions of the flocks were set to correspond to 01/01/2006, which is the real-world time when this specific simulation is logically set to commence. The dispersion of poultry population in different continents has been approximated to circular regions with even density in the Eco-description [16, 25]. Poultry data has been collated from statistics published by national organizations and government databases [16]. Currently, we have represented human population, as reported by U.S. Census Bureau [17], in all 26 major metropolitan areas of the United States, approximated to circular regions. However, the Eco-description can be readily extended to include other parts of the world. Note that to the best of our knowledge, it is the most comprehensive model of its kind reported to date.

### VERIFICATION OF SEARUMS

Having developed the Eco-description, we verified the validity of the Eco-description by performing extensive simulations with the initial source of infection set to outbreak in Indonesia [5]. We established the validity of the Eco-description and SEARUMS by confirming that the timing and chronology of several outbreaks observed in the simulations correlate with significant real-world incidents as reported by WHO [5]. The data in Table 2 presents a comparison of real-world and simulated outbreaks. The data is presented for some of the significant, initial avian influenza outbreaks and not for repeated outbreaks that occur in these regions. Note that deviations of $\pm 2$ weeks is expected due to approximation of migration dates. In addition, deviations in dates also occur because the Eco-description does not include all types of migratory waterfowl flocks but only the high risk species. However, the sufficiently close concordance between simulated and real-world outbreaks establishes validity and effectiveness of SEARUMS. Moreover, it significantly increases confidence in inferences drawn from the simulation. Currently, a variety of case studies using SEARUMS are already underway to predict and avert a

TABLE 1. Various agent instances used to develop the Eco-description used for experiments. Entries prefixed with * indicate high risk waterfowl species [27].

| Description of AgentType | Number of Agents | Total Population | #Countries in Migration Path |
|---|---|---|---|
| Bar-Tailed Godwit | 4 | 40,000 | 18 |
| Canada Goose | 16 | 231,700 | 5 |
| Common Crane | 9 | 225,000 | 21 |
| *Eurasian Widgeon | 3 | 1,296,000 | 17 |
| Great Knot | 3 | 231,000 | 8 |
| *Mallard | 1 | 5,000 | 1 |
| Razorbill | 1 | 148,000 | 4 |
| *Red-Breasted Goose | 1 | 44,000 | 4 |
| Red-crowned Crane | 1 | 15,000 | 4 |
| Siberian Crane | 3 | 30,000 | 12 |
| Yellow-Billed Duck | 2 | 20,000 | 8 |
| Total waterfowl flocks | 44 | 2,285,700 | 40 |
| Total Poultry flocks | 121 | 12,280,000,000 | 32 |
| Total Human groups | 26 | 125,000,000 | 1 |
| **Total** | **191** | **12,407,285,700** | **40** |

TABLE 2. Comparison of chronology of significant Real-world outbreaks with simulated outbreaks

| Incident | Real-world Date | Simulated Date | Error (Days) |
|---|---|---|---|
| Outbreak in Indonesia | 23-Jan-06 | 1-Jan-06 | 22 |
| Infection in Iraq/Iran | 1-Mar-06 | 25-Mar-06 | 24 |
| Infection in China | 27-Apr-06 | 2-Apr-06 | -25 |
| Infection in Egypt | 11-Oct-06 | 14-Sep-06 | -27 |

pandemic.

### Scalability and Performance Evaluation

One of the important objectives underlying the design of SEARUMS is to facilitate rapid analysis of epidemiology of avian influenza. Accordingly, the validated Eco-description was also used to conduct scalability and performance evaluation of SEARUMS using three different platforms. The first platform was a Sun Netra-T12 SMP workstation with eight 1.2 GHz SPARCv9 processors with 16 gigabytes of RAM running Solaris 9. The second platform was a conventional personal computer (PC) running Windows XP (32-bit). The PC had a dual-core, 64-bit Turion processor running at 2 GHz with 2 GB of memory. The third platform was the same PC but running Linux (Fedora Core). Specifically, the PC was setup to a dual-boot configuration enabling it to run either Windows XP or Linux. These simulations were conducted by running SEARUMS

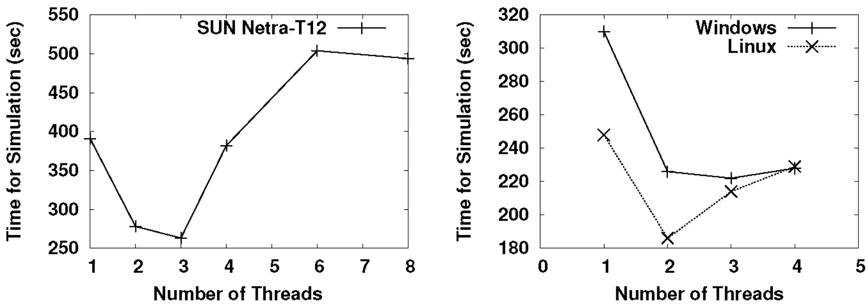

(a) Sun Netra-T12 (b) PC (Windows & Linux)

**FIGURE 2.** Influence of number of threads on wall-clock time for simulation on different hardware platforms and operating systems.

in batch mode, without any GUI overhead to ensure emphasis on core performance.

The graph in Figure 2 plots the wall-clock time taken to complete the simulation using a varying number of threads on the aforementioned platforms. As shown by the curves, initially the time taken for simulation reduces as the number of threads are increased. The wall-clock time decreases because the threads run in parallel on the multi-processor/multi-core machines and rapidly process concurrent events. The initial performance improvement highlights SEARUMS' scalability. However, the performance decreases as the number of threads are further increased. The deterioration occurs because the Eco-description used in this experiment does not have sufficient, inherent concurrency to leverage the available compute power. On the other hand, larger models with more concurrency will benefit from the scalable design.

These experiments highlight the portability, scalability, and performance aspects of SEARUMS design. Simulations involving more than 20 million events complete within 4 minutes in optimal cases demonstrating that SEARUMS enables rapid epidemiological analysis of avian influenza. Furthermore, the experiments provide empirical evidence that the design goals of SEARUMS have been successfully achieved.

### Case Study: Spread and Impact to United States

In this case study, we analyzed the potential impact of avian influenza to poultry farming in the United States using the validated Eco-description presented earlier. The study was conducted using a number of bio-simulations with three different experimental groups of migrating waterfowl. The three flocks were chosen based on their close proximity to known primary sites of disease outbreaks. The initial infection in each experimental group was varied for analysis. In all these experiments, the initial conditions were seeded corresponding to January 1, 2006 and the simulations were run for a period of 5 years.

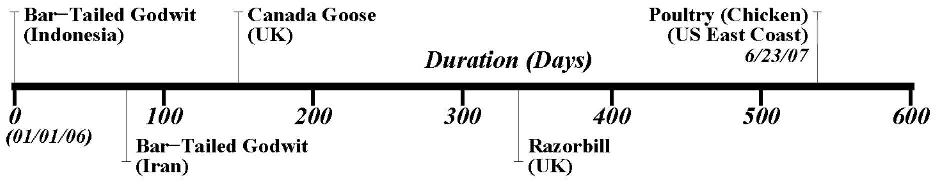

**FIGURE 3.** Timelines for H5N1 infection spread from experiment logically set to start on 01/01/2006.

Figure 3 illustrates one of the trans-Atlantic transmission pathways to the continental United States. We observed that the spread was determined by migratory pathways and timelines of different species of waterfowl rather than initial infection percentages. One of the notable observations is that our experiments correctly predicted an outbreak in the United Kingdom [4]. The graph in Figure 4 presents the impact of avian influenza outbreaks on poultry population in continental United States. Decrease in poultry population corresponds to H5N1 induced death and culling of birds to control the disease. Increase in poultry population reflects regeneration of poultry flocks after an outbreak. As illustrated by the graph, infections in poultry also follow a cyclic pattern that correlate with annual migration of waterfowl. The mortality figures can be translated to corresponding dollar figures for financial analysis.

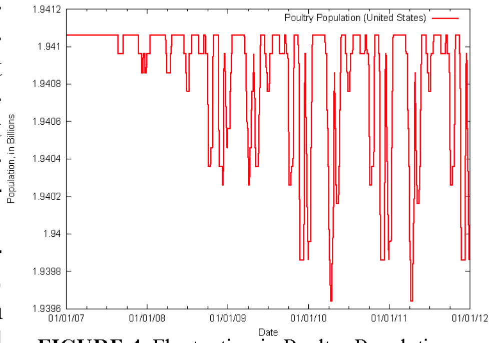

**FIGURE 4.** Fluctuation in Poultry Population

## CONCLUSIONS

The application of computational evolutionary methodology to analyze the epidemiology of avian influenza was presented. Specifically, the practical issues involved in applying this methodology using an eco-modeling and bio-simulation environment called SEARUMS were discussed. The evolutionary epidemiological model of avian influenza was developed and empirically verified. The verification experiments highlight that the proposed methodology closely reflects real world scenarios. The bio-simulations conducted using SEARUMS provide good forecast of time lines and epicenters of outbreaks. Furthermore, it was observed that outbreaks follow a cyclical, annual pattern. This new, comprehensive knowledge gained about epidemiology of avian influenza is invaluable to combat outbreaks and prevent a pandemic. We are optimistic that the proposed software environment will enable mankind to strategically invest precious time and resources to combat diseases like avian influenza, minimize their impacts on human life and global economy.
